# *Staphylococcus aureus* Impairs the Function of and Kills Human Dendritic Cells via the LukAB Toxin

**DOI:** 10.1128/mBio.01918-18

**Published:** 2019-01-02

**Authors:** Evelien T. M. Berends, Xuhui Zheng, Erin E. Zwack, Mickaël M. Ménager, Michael Cammer, Bo Shopsin, Victor J. Torres

**Affiliations:** a Department of Microbiology, New York University School of Medicine, New York, New York, USA; b Molecular Pathogenesis Program, The Kimmel Center for Biology and Medicine of the Skirball Institute, New York University School of Medicine, New York, New York, USA; c Microscopy Laboratory, Division of Advanced Research Technologies, New York University School of Medicine, New York, New York, USA; d Department of Medicine, Division of Infectious Diseases, New York University School of Medicine, New York, New York, USA; University of Illinois at Chicago

**Keywords:** MRSA, *Staphylococcus aureus*, antigen-presenting cells, dendritic cells, immune system, leukocidin, pore-forming toxins, toxin

## Abstract

Staphylococcus aureus is a human pathogen responsible for high morbidity and mortality worldwide. Recurrent infections with this bacterium are common, suggesting that S. aureus thwarts the development of sterilizing immunity. S. aureus strains that cause disease in humans produce up to five different bicomponent toxins (leukocidins) that target and lyse neutrophils, innate immune cells that represent the first line of defense against S. aureus infections. However, little is known about the role of leukocidins in blunting adaptive immunity. Here, we explored the effects of leukocidins on human dendritic cells (DCs), antigen-presenting cells required for the development of adaptive immunity. Using an *ex vivo* infection model of primary human monocyte-derived dendritic cells, we found that S. aureus, including strains from different clonal complexes and drug resistance profiles, effectively kills DCs despite efficient phagocytosis. Although all purified leukocidins could kill DCs, infections with live bacteria revealed that S. aureus targets and kills DCs primarily via the activity of leukocidin LukAB. Moreover, using coculture experiments performed with DCs and autologous CD4^+^ T lymphocytes, we found that LukAB inhibits DC-mediated activation and proliferation of primary human T cells. Taken together, the data determined in the study reveal a novel immunosuppressive strategy of S. aureus whereby the bacterium blunts the development of adaptive immunity via LukAB-mediated injury of DCs.

## Abstract (precis)

Antigen-presenting cells such as dendritic cells (DCs) fulfill an indispensable role in the development of adaptive immunity by producing proinflammatory cytokines and presenting microbial antigens to lymphocytes to trigger a faster, specific, and long-lasting immune response. Here, we studied the effect of Staphylococcus aureus toxins on human DCs. We discovered that the leukocidin LukAB hinders the development of adaptive immunity by targeting human DCs. The ability of S. aureus to blunt the function of DCs could help explain the high frequency of recurrent S. aureus infections. Taken together, the results from this study suggest that therapeutically targeting the S. aureus leukocidins may boost effective innate and adaptive immune responses by protecting innate leukocytes, enabling proper antigen presentation and T cell activation.

## INTRODUCTION

Staphylococcus aureus is an important opportunistic Gram-positive pathogen that causes infections in humans (1). Around 30% of the human population is asymptomatically colonized with S. aureus (2). However, when S. aureus manages to become invasive, it causes a wide array of serious infections. With no vaccine currently available (3) and with the increasing levels of multidrug-resistant methicillin-resistant S. aureus (MRSA) strains (4), S. aureus infections pose a serious public health threat. MRSA has plagued the hospitals for decades and is now frequently recovered from seemingly healthy individuals owing to the emergence of community-associated MRSA (CA-MRSA). CA-MRSA infections caused by clone USA300, the predominant cause of community-acquired skin infections in the United States (5, 6), frequently recur, indicating that primary infection does not induce protective immunity.

A key feature of S. aureus that facilitates its pathogenic lifestyle is the production of a large array of virulence factors that thwart the immune system (7, 8). An important group of these virulence factors consists of the bicomponent pore-forming leukocidins (here referred to collectively as “leukocidins”) (9, 10). S. aureus clinical isolates, including USA300 (11), produce up to five different leukocidins: leukocidin ED (LukED), Panton-Valentine leukocidin (PVL), leukocidin AB (LukAB, also known as LukGH), and γ-hemolysins AB and CB (HlgAB and HlgCB) (9, 10). Leukocidins consist of two subunits (denoted S and F) that oligomerize to form membrane-spanning pores that lyse target cells. These toxins target a wide array of immune cells (9, 10), the most extensively studied of which are the neutrophils, representing critical components of the initial immune defense against bacteria (7). Initial binding of the toxin occurs via recognition of leukocyte receptors, which dictate the cell specificity exhibited by these toxins (10). Additionally, the specific targeting of human receptors but not of the counterpart receptors in mice leads to human-specific tropism that hampers research of these toxins *in vivo* (10).

While the activity of leukocidins against human neutrophils, monocytes, and macrophages has been well documented (10), the effects of these toxins on dendritic cells (DCs), which are considered the most important and efficient antigen-presenting cells within the immune system (12, 13), remain to be fully defined. Bridging innate immunity and adaptive immunity, DCs fulfill an indispensable role in the development of durable immune protection by producing proinflammatory cytokines and presenting microbial antigens to lymphocytes (12). However, the details of S. aureus-human DC interactions and the mechanisms employed by the bacterium to subvert these important immune cells remain incompletely defined (14–17).

In this study, we characterized the interaction between S. aureus and human monocyte-derived DCs. Our data indicate that S. aureus targets and kills DCs, an effect mediated primarily by the LukAB leukocidin. Moreover, we demonstrate that by both directly killing and dampening levels of antigen presentation molecules on the surface of DCs, LukAB impairs DC-mediated activation of CD4^+^ T lymphocytes. Collectively, our data suggest that targeting DCs could facilitate S. aureus pathogenesis by blunting the development of adaptive immunity.

## RESULTS

### S. aureus kills human DCs independently of clonal complex or drug resistance.

**FIG 1:**
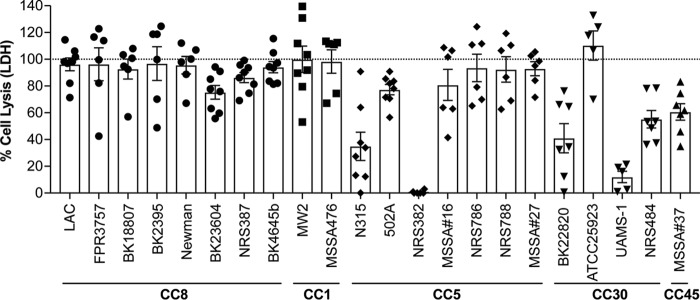
S. aureus targets and kills human DCs. Monocyte-derived DCs were infected with a panel of clinical isolates from different clonal complexes, including MSSA and MRSA strains. Cells were infected at an MOI of 10 for 2 h. As a measure of cell lysis, the release of LDH was monitored (% Cell Lysis) and normalized to 100% cell lysis with 0.05% Triton X-100. Each data point represents an individual human donor; the bars indicate overall means ± standard errors of the means (SEM); *n* = 5 to 8 donors.

To study S. aureus-DC interactions, we generated human monocyte-derived DCs (MDDCs or DCs) and infected them with a collection of S. aureus isolates from different clonal complexes (CC). Our panel comprised methicillin-sensitive S. aureus (MSSA) and methicillin-resistant S. aureus (MRSA) strains, including strains associated with hospital-acquired and community-acquired infections (Table 1). Overall, these experiments demonstrated that, in general, S. aureus kills DCs independently of the clonal complex, antibiotic resistance, or the type of clinical infection from which isolates were obtained (Fig. 1; see also Table 1).

**TABLE 1:** *S. aureus* isolates and strains used in study^*a*^

Strain	Background	Description	Reference or source
VJT 12.61	USA300	WT LAC	60
VJT 20.06	USA300	WT FPR3757	61
VJT 15.36	USA300	WT BK18807	25
VJT 10.21	USA500	WT BK2395	62
VJT 1.01	Newman	WT Newman MSSA	20
VJT 15.46	ST239	WT BK23604 (BS991)	Shopsin laboratory
VJT 21.06	USA800	WT NRS387	NARSA (25)
VJT 5.81	CC8	WT BK4645b MSSA	63
VJT 4.79	USA400	WT MW2 CA-MRSA	64
VJT 50.06	CC1	WT MSSA476	65
VJT 50.03	CC5	WT N315 HA-MRSA	66
VJT 50.04	USA700	WT 502A	67
VJT 21.02	USA100	WT NRS382	NARSA
VJT 23.05	USA100	WT MSSA#16	40
VJT 35.72	USA100	WT NRS786	NARSA
VJT 35.74	USA100	WT NRS788	NARSA
VJT 38.02	USA100	WT MSSA#27	40
VJT 15.44	CC30	WT BK22820 phage-type 80/81 pandemic strain (BS992)	Shopsin laboratory
VJT 50.09	CC30	WT ATCC 25923	ATCC
VJT 2.59	USA200	WT UAMS-1	68
VJT 21.08	USA1100	WT NRS484	NARSA (25)
VJT 38.11	USA600	WT MSSA#37	40
VJT 14.26	USA300	Δ*lukAB* LAC	20
VJT 16.39	USA300	Δ*lukAB* BK18807	20
VJT 11.39	USA400	Δ*lukAB* MW2	20
VJT 22.31	Newman	Δ*lukAB* Newman	20
VJT 11.36	CC8	Δ*lukAB* BK4645b	20
VJT 15.78	USA300 LAC	WT (AH1263)	69
VJT 47.15	USA300 LAC	*hlgACB*::*tet lukED*::*kan pvl*::*spec* Δ*lukAB* (ΔΔΔΔΔ USA300)	70
VJT 44.10	USA300 LAC	*pvl*::*spec*	71
VJT 30.01	USA300 LAC	*lukED*::*kan*	71
VJT 29.98	USA300 LAC	*hlgACB*::*tet*	70
VJT 23.52	USA300 LAC	Δ*lukAB*	11
VJT 38.84	USA300 LAC	*hlgACB*::*tet lukED*::*kan pvl*::*spec*	70
VJT 49.33	USA300 LAC	WT pOS1-*P_sarA_*-*SOD*-*RBS*-sGFP (Cm^r^)	This study
VJT 49.34	USA300 LAC	*hlgACB*::*tet lukED*::*kan pvl*::*spec* Δ*lukAB* pOS1-*P_sarA_*-*SOD*-*RBS*-sGFP (Cm^r^)	This study
VJT 49.35	USA300 LAC	Δ*lukAB* pOS1-*P_sarA_*-*SOD*-*RBS*-sGFP (Cm^r^)	This study
VJT 49.36	USA300 LAC	*hlgACB*::*tet lukED*::*kan pvl*::*spec* pOS1-*P_sarA_*-*SOD*-*RBS*-sGFP (Cm^r^)	This study
VJT 26.87	USA300 LAC	pXEN1-lux, luciferase reporter promoterless control (Cm^r^)	11
VJT 26.89	USA300 LAC	pXEN1-p*lukAB*-lux, *lukAB* promoter driving expression of *lux* operon (Cm^r^)	11
VJT 26.91	USA300 LAC	pXEN1-p*lukSF-PV-*lux, *pvl* promoter driving expression of *lux* operon (Cm^r^)	11
VJT 26.92	USA300 LAC	pXEN1-p*hlgCB-*lux, *hlgCB* promoter driving expression of *lux* operon (Cm^r^)	11
VJT 26.93	USA300 LAC	pXEN1-p*hlgA-*lux, *hlgA* promoter driving expression of *lux* operon (Cm^r^)	11
VJT 26.95	USA300 LAC	pXEN1-p*lukED-*lux, *lukED* promoter driving expression of *lux* operon (Cm^r^)	11
VJT 31.57	Newman	Δ*lukED* Δ*hlgACB*::*tet* Δ*lukAB*::*spec* Δ*hla*::*ermC* (ΔΔΔΔΔ Newman)	59

a WT, wild type; HA-MRSA, hospital-acquired S. aureus; Cm^r^, chloramphenicol resistance; NARSA, Network on Antimicrobial Resistance in *S. aureus*; ATCC, American Type Culture Collection.

### Leukocidins directly kill human DCs.

**FIG 2:**
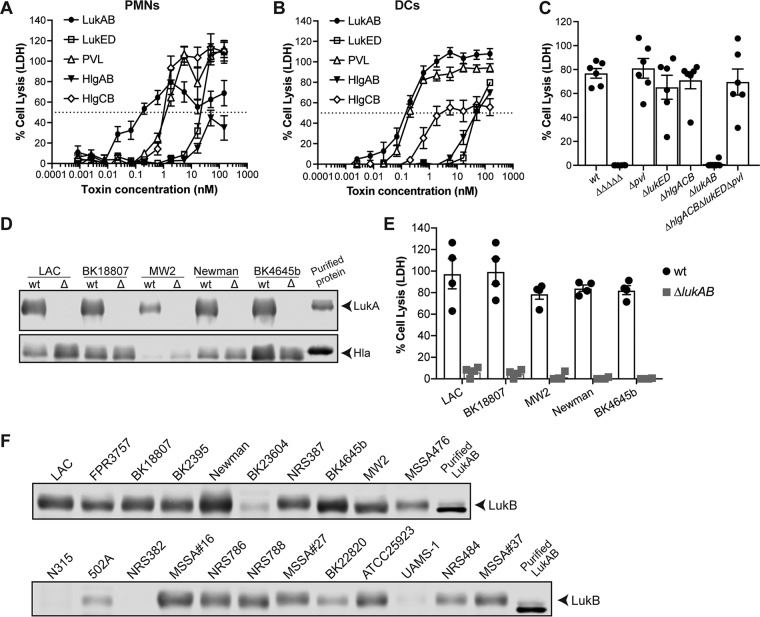
LukAB is responsible for killing DCs during S. aureus infection. (A and B) Viability of primary human PMNs (A) and monocyte-derived DCs (B) after exposure to purified leukocidins was analyzed by detecting the release of LDH. Bars indicate means ± SEM, with *n* = 7 donors. (C) Infection of monocyte-derived DCs for 2 h at an MOI of 10 with a panel of isogenic mutant strains in the USA300 AH-LAC background. Each data point represents an individual human donor; the bars indicate means of percentages of cell lysis ± SEM as measured by LDH release; *n* = 6 donors. (D) The presence and absence of LukAB in culture supernatants of a panel of isogenic mutants were evaluated by immunoblotting with an anti-LukA antibody (top panel). In addition, the presence of alpha-toxin (Hla) was analyzed to verify that the *lukAB* mutation did not interfere with the production of other S. aureus toxins. (E) Infection of monocyte-derived DCs for 2 h at an MOI of 10 with isogenic wt and Δ*lukAB* bacteria from different MSSA and MRSA backgrounds. Each data point represents an individual human donor; the bars indicate means of percentages of cell lysis ± SEM as measured by LDH release; *n* = 4 donors. (F) The presence of LukAB in culture supernatants of S. aureus clinical isolates was evaluated by immunoblotting with an anti-LukB antibody.

Among the virulence factors produced by S. aureus, the leukocidins are prime candidates for DC cytolysins as these potent toxins are known to target other human leukocytes during S. aureus-host cell interactions (10). Since all leukocidins target primary human neutrophils (also known as polymorphonuclear leukocytes [PMNs]) (18), we first compared the levels of susceptibility of human DCs and PMNs to purified leukocidins. As expected, all the leukocidins lysed human PMNs (18), albeit with different potencies (Fig. 2A). We found that all the leukocidins also killed DCs, with LukAB and PVL being the most lytic (Fig. 2B).

### S. aureus kills DCs via LukAB.

Next, we examined if the leukocidins are responsible for killing DCs during infection with live S. aureus. To this end, we used a panel of isogenic mutants in a representative CA-MRSA USA300 strain background (19). The wild-type (wt) USA300 strain potently killed DCs, whereas a mutant lacking all the leukocidins (Δ*hlgACB* Δ*lukED* Δ*pvl* Δ*lukAB* USA300 [ΔΔΔΔΔ USA300]) was unable to lyse those cells (Fig. 2C). We next tested isogenic USA300 mutants that lacked each of the individual leukocidins. These experiments identified LukAB as the leukocidin responsible for USA300-mediated targeting and killing of DCs during infection (Fig. 2C), as the deletion of *lukAB* mirrored the lack of cytotoxicity exhibited by the isogenic strain lacking all the toxins. Consistent with this observation, an isogenic mutant lacking all leukocidins except LukAB (mutant Δ*hlgACB* Δ*lukED* Δ*pvl*) exhibited cytolytic activity similar to that shown by the wt strain (Fig. 2C).

To further investigate the role of LukAB in killing DCs, we used a collection of isogenic wt or Δ*lukAB* MRSA and MSSA strains (Table 1) (20). Western blot analyses of these strains verified the presence or absence of LukAB. All the strains produced alpha-toxin (Hla), which was used as a positive control (Fig. 2D). Infection of DCs with this diverse collection of strains further established the role of LukAB in killing human DCs (Fig. 2E). Since most of the tested clinical isolates were cytotoxic toward DCs (Fig. 1), we next analyzed the supernatants of these strains by Western blotting to correlate LukAB levels with their cytotoxic activity. As expected, LukAB was produced by all the highly cytotoxic strains whereas lower levels were detected in the supernatant of strains that were less cytotoxic toward DCs (e.g., strains N315, UAMS-1, and NRS382) (Fig. 2F).

### PMNs and DCs are equally susceptible to LukAB-mediated killing during S. aureus infection.

**FIG 3:**
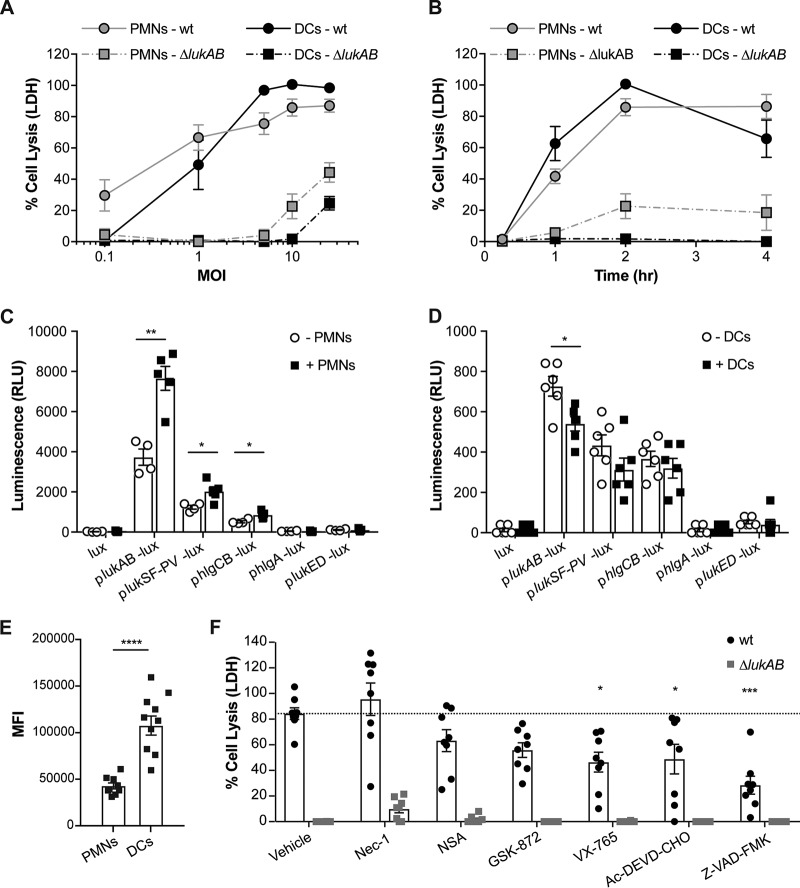
LukAB-mediated killing of DCs during S. aureus infection is associated with increased CD11b levels and activation of apoptosis. (A) A direct comparison of the levels of infection of PMNs and monocyte-derived DCs with isogenic wt or Δ*lukAB* USA300 AH-LAC. Cell lysis was measured via LDH release after 2 h of infection at MOIs of 0.1, 1, 5, 10, and 25. (B) Infection of PMNs and monocyte-derived DCs with wt and Δ*lukAB* bacteria at an MOI of 10 for 15 min, 1, 2, and 4 h. Values in panels A and B are plotted as means ± SEM; *n* = 4 donors. (C and D) Leukocidin promoter activity was measured by luminescence of wt bacteria harboring plasmids of leukocidin promoter sequences fused to the luciferase gene in the absence or presence of PMNs (C) and monocyte-derived DCs (D). Each data point represents an individual human donor; the bars indicate means ± SEM; *n* = 5-6 donors. Data were analyzed using an unpaired, two-tailed Student's *t* test. RLU, relative light units. (E) Semiquantitative levels of total CD11b (geometric mean fluorescence intensity [MFI]) on both PMNs and monocyte-derived DCs from the same donors were measured by flow cytometry; *n* = 9 donors. The results were analyzed using a paired, two-tailed Student's *t* test. (F) Monocyte-derived DCs were pretreated with 2% DMSO (Vehicle), 200 µM necrostatin-1 (Nec-1), 100 µM necrosulfonamide (NSA), 50 µM GSK-872, 200 µM VX-765, 200 µM Ac-DEVD-CHO, or 200 µM Z-VAD-FMK and were then infected with wt or Δ*lukAB* bacteria at an MOI of 5. Cell lysis was measured via LDH release 2 h postinfection. The dotted horizontal line represents the mean percentages of cell lysis of wt-infected, vehicle-treated cells. Each data point represents an individual human donor; bars indicate means ± SEM; *n* = 8 donors. *P* values against the vehicle control were determined using one-way analysis of variance (ANOVA) and Dunnett’s multiple-comparison tests. Asterisks indicate statistical differences with *P* values as follows: *, *P* < 0.05; **, *P* < 0.01; ***, *P* < 0.001; ****, *P* < 0.0001.

We next compared the susceptibility of both PMNs and DCs to infection with USA300 wt and Δ*lukAB* bacteria at various multiplicities of infection (MOIs) for 2 h (Fig. 3A) or at different time points postinfection with an MOI of 10 (Fig. 3B). These results highlight that human PMNs and DCs are similarly susceptible to USA300-mediated killing. Regardless, we observed that LukAB is the dominant toxin that mediates killing of both human phagocytes irrespective of the bacterial inoculum or infection time (Fig. 3A and B).

Previously, it was shown that human PMNs induce the expression and production of LukAB (11, 21, 22), which is associated with the dominant role of this toxin in infections using primary human PMNs (11, 20, 23). Thus, we tested whether DCs are also susceptible to S. aureus infection because they induce *lukAB* expression. To address this, we utilized USA300 reporter strains where each individual leukocidin promoter is fused to the luciferase operon from Photorhabdus luminescens (11). Consistent with previous findings, exposure of human PMNs to USA300 induced upregulation of several leukocidin promoters, predominantly *lukAB* (Fig. 3C). Interestingly, we found that exposure of USA300 to DCs does not increase the activation of the leukocidin promoters. In fact, the *lukAB* promoter activity was slightly decreased in the presence of DCs (Fig. 3D). These results suggest that even though DCs exhibit susceptibility to USA300-mediated killing similar to that exhibited by PMNs, their susceptibility to LukAB is not due to induction of the toxin during infection of these cells.

LukAB targets CD11b for lysis of human PMNs and monocytes (24, 25). Thus, we compared the levels of CD11b on the surface of PMNs and DCs by using antibodies and flow cytometry to detect CD11b on both phagocytes. We found that DCs have over 2.5-fold the amount of surface CD11b shown by PMNs (mean fluorescence intensity [MFI], 42,285 ± 3,189 versus 106,991 ± 10,309) (Fig. 3E). Thus, the susceptibility of DCs to LukAB is associated with higher levels of the toxin receptor on these cells.

### S. aureus kills DCs through activation of caspase-dependent pathways.

S. aureus and its toxins activate a wide range of cell death pathways from apoptosis to pyroptosis and necroptosis in human neutrophils, monocytes, and macrophages (25–31). Thus, we next set out to elucidate how LukAB induces death in DCs during S. aureus infection. DCs were treated with inhibitors for enzymes known to play important roles in apoptosis (Z-VAD-FMK and Ac-DEVD-CHO), pyroptosis (VX-765), and necroptosis (Nec-1, NSA, and GSK-872) for 30 min prior to infecting the DCs with either USA300 wt or Δ*lukAB* bacteria. We observed a significant decrease in LukAB-mediated cell death when DCs were treated with the pan-caspase inhibitor Z-VAD or specific inhibitors against caspase-3 (Ac-DEVD-CHO) and against caspase-1 and caspase-4 (VX-765). In contrast, no significant decrease in cell death was observed when DCs were treated with inhibitors for RIPK1 (Nec-1), MLKL (NSA), or RIPK3 (GSK-872) (Fig. 3F). These data suggest that DCs die via caspase-mediated apoptosis and pyroptosis during S. aureus infection.

### S. aureus lyses DCs independently of the level of phagocytosis.

**FIG 4:**
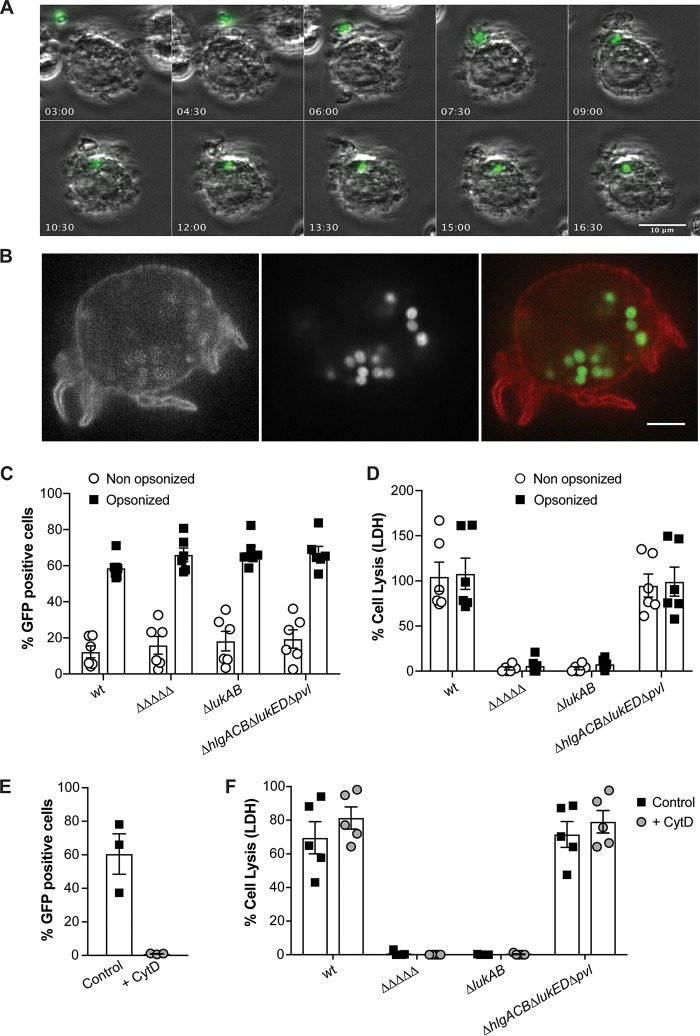
S. aureus kills DCs independently of the level of phagocytosis. (A) Time-lapse imaging of opsonized, fluorescent Δ*hlgACB* Δ*lukED* Δ*pvl* Δ*lukAB* (ΔΔΔΔΔ) USA300 (green) taken up by monocyte-derived DCs (gray). The bar represents 10 μm. (B) Spatial localization of fluorescent ΔΔΔΔΔ USA300 (green) taken up by monocyte-derived DCs (red). The bar indicates 5 μm. (C) Uptake of isogenic GFP-expressing USA300 strains by monocyte-derived DCs. Bacteria were preincubated with media (nonopsonized) or human serum (opsonized). GFP fluorescence of the DCs was analyzed by flow cytometry. Each data point represents an individual human donor; bars indicate means ± SEM; *n* = 6 donors. (D) Infection of monocyte-derived DCs with nonopsonized and opsonized USA300 at an MOI of 10 for 2 h, after which the release of LDH release was measured. Each data point represents an individual human donor; bars indicate means ± SEM; *n* = 6 donors. (E) Uptake of wt USA300 in the presence or absence of 10 μg/ml cytochalasin D (CytD). Monocyte-derived DCs were treated with 40 µg/ml lysostaphin to kill bacteria sticking to the outer side of the cells. Each data point represents an individual human donor; bars indicate means ± SEM; *n* = 3 donors. (F) Infection of monocyte-derived DCs with USA300 in the presence or absence of 10 μg/ml CytD, after which LDH release was measured. Each data point represents an individual human donor; bars represent means ± SEM; *n* = 5 donors; MOI = 10.

DCs are phagocytes that engulf microbes to promote antigen presentation. Therefore, we next analyzed the interaction of DCs and S. aureus using time-lapse microscopy. To track bacteria, we opsonized fluorescent USA300 with complement-containing human serum to promote uptake. Upon infection of DCs, bacteria were captured and internalized within 20 min (Fig. 4A and B).

We next studied the USA300-DC interaction in greater detail by flow cytometry. For these experiments, fluorescent USA300 isogenic strains were used to infect DCs in the presence or absence of complement-containing human serum. We observed that opsonization promoted bacterial uptake (10% without serum to 60% with serum) (Fig. 4C). Of note, the uptake of USA300 was independent of the presence or absence of leukocidins (Fig. 4C).

We took advantage of the altered phagocytosis observed in comparisons of opsonized and nonopsonized bacteria to determine whether S. aureus preferentially kills DCs from outside the cells (extracellular) or from within the cells (intracellular). These experiments revealed that while opsonization influences bacterial uptake, it did not alter the lysis of the DCs (Fig. 4D). These findings were confirmed in assays where bacterial uptake was prevented by using cytochalasin D (Fig. 4E and F), an agent that blocks actin polymerization and, thus, phagocytosis. Collectively, these data demonstrate that S. aureus is effectively taken up by DCs and that the bacteria kill these independently of the level of phagocytosis in a LukAB-dependent manner.

### LukAB blocks DC-mediated activation and proliferation of human T cells.

**FIG 5:**
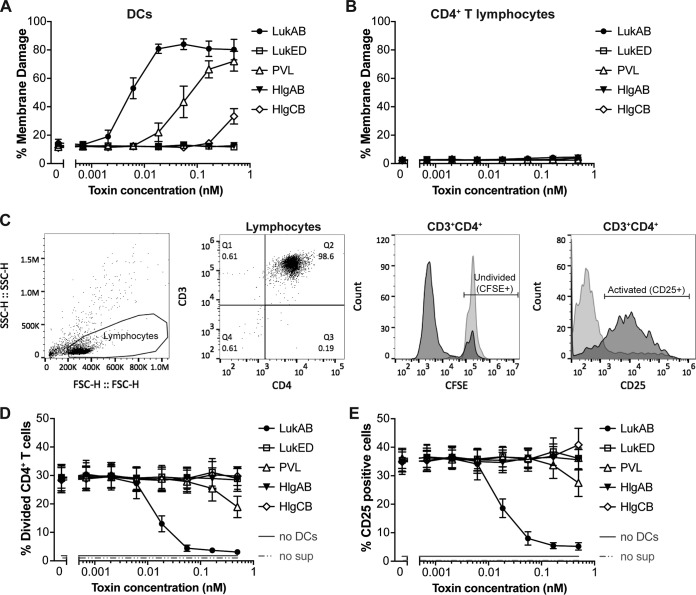
Purified LukAB dampens DC-mediated activation of human CD4^+^ T lymphocytes. (A and B) Viability of monocyte-derived DCs (A) and CD4^+^ T cells (B) upon exposure to low concentrations of leukocidins over a period of 24 h, as determined by the use of a membrane-impermeative viability dye (eFluor 450). Data are plotted as mean values ± SEM; *n* = 5 donors. (C) Gating strategy to identify the CD4^+^ T cells in the total lymphocyte population (two left plots). The right two plots indicate how cell division (CFSE) and CD4^+^ T cell activation (CD25^+^) are monitored. (D and E) CD4^+^ T cell division (% Divided CD4^+^ cells) (D) and activation (% CD25 positive cells) (E) in the presence of a concentration gradient of different leukocidins. Data are plotted as mean values ± SEM; *n* = 6 donors. no sup, no supplementation.

To determine whether LukAB-mediated targeting of DCs inhibits the initiation of adaptive immunity, we assayed the activation and proliferation of primary human CD4^+^ T cells by DCs. First, the lytic effect of leukocidins on DCs was assessed at 24 h after toxin exposure (in contrast to intoxication for 1 h as performed in the experiments whose results are presented in Fig. 2B) using significantly lower (>100-fold) concentrations of toxin. Consistent with data obtained using high doses of toxin, DCs suffered membrane damage after 24 h of exposure to low doses of LukAB (≥0.006 nM) and PVL (≥0.06 nM) (Fig. 5A). As a control, we also exposed CD4^+^ T cells to the leukocidins and found that those cells remained intact under these conditions (Fig. 5B).

We then performed experiments in which DCs were cocultured with autologous carboxyfluorescein succinimidyl ester (CFSE)-labeled CD4^+^ T cells and stimulated with S. aureus cell-free supernatant from a strain lacking all the leukocidins. Of note, S. aureus supernatants contain a mix of antigens as well as superantigens that trigger nonspecific T cell activation. To determine the effect of toxins on T cell activation, purified leukocidins (or control buffer) was exogenously added to the coculture and the proliferation and activation of T lymphocytes were analyzed by flow cytometry. CFSE signal was used as a measure of lymphocyte proliferation, and the upregulation of CD25 was used as a marker of lymphocyte activation (Fig. 5C). CD4^+^ T cells were not activated in the absence of DCs or S. aureus supernatant, confirming that it was a DC- and S. aureus supernatant-dependent response (Fig. 5D and E). After 3 days of coculture, ∼30% of the T cells divided (Fig. 5D), which was accompanied by ∼35% of the cells upregulating CD25 (Fig. 5E). These responses were blunted when DCs were exposed to LukAB (Fig. 5D and E).

### S. aureus blunts the activation of CD4^+^ T lymphocytes by targeting DCs via LukAB.

**FIG 6:**
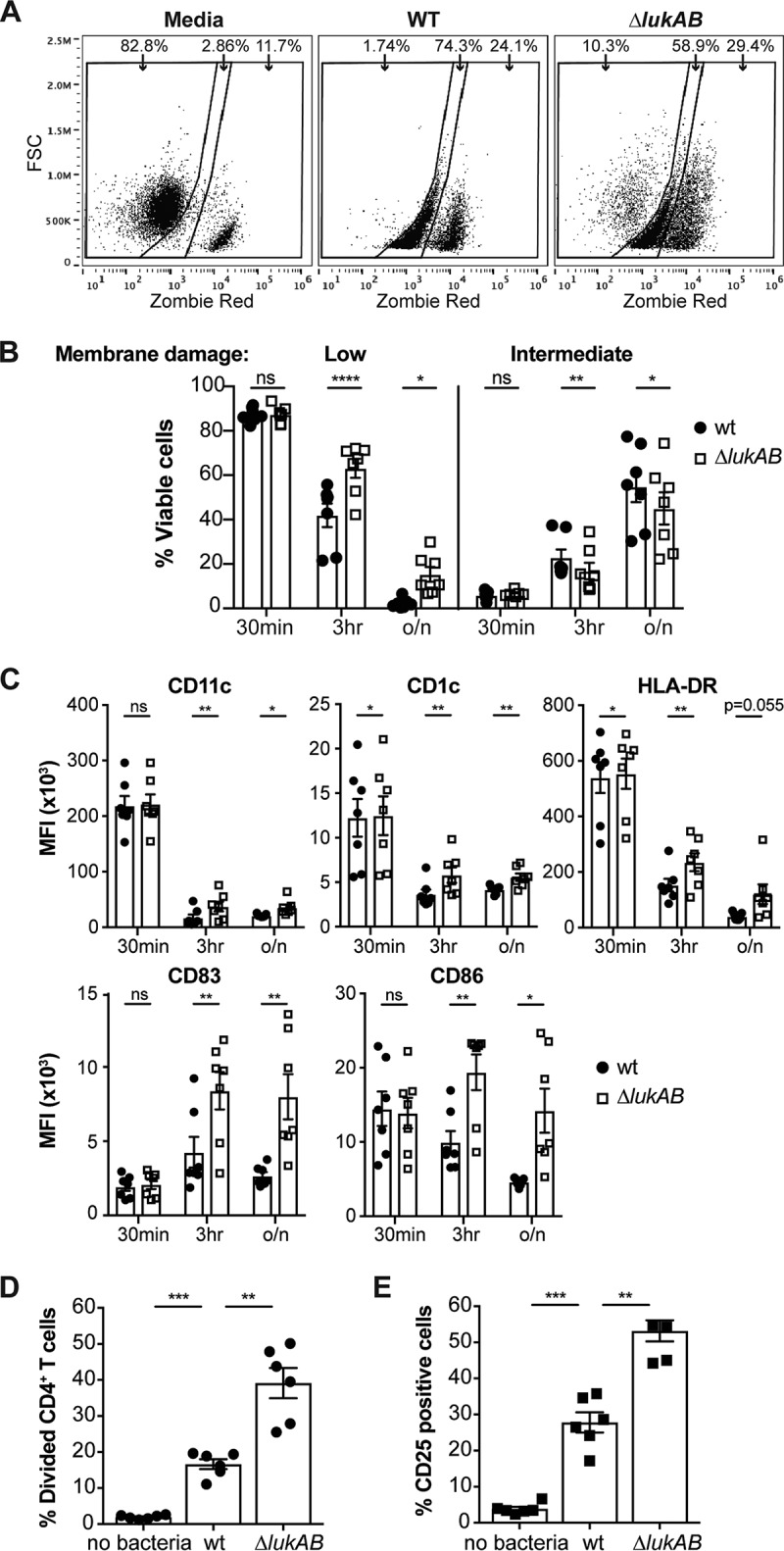
S. aureus inhibits DC-meadiated activation of human CD4^+^ T lymphocytes via LukAB. (A) Gating strategy to define membrane damage in DCs upon infection with wt or Δ*lukAB* USA300 at an MOI of 5. Membrane damage was measured by the incorporation of Zombie Red viability dye. From left to right, the three gates indicate low-level/negligible, intermediate-level, and high-level membrane damage. The percentage of DCs in each gate from a representative donor after overnight infection is shown. FSC, forward scatter. (B) Quantification of the percentages of cells in low-level and intermediate-level membrane damage gates at 30 min postinfection, 3 h postinfection, and after one night (overnight [o/n]) with wt or Δ*lukAB* USA300 for all donors. (C) Geometric mean fluorescence intensity (MFI) levels were measured for CD11c, CD1c, HLA-DR, CD83, and CD86 on the surface of DCs infected with wt or Δ*lukAB* USA300 by flow cytometry. In panels B and C, each data point represents an individual human donor, bars are plotted as mean values ± SEM; *n* = 7 donors. The results were analyzed using a paired, two-tailed Student's *t* test. (D and E) CD4^+^ T cell proliferation (% Divided CD4^+^ cells) (D) and activation (% CD25 positive cells) (E) after incubation with DCs that were left unexposed (no bacteria) or infected with wt or Δ*lukAB* USA300 at an MOI of 5. CD4^+^ T cell proliferation and activation levels were measured 3 days postcoculturing with DCs. Each data point represents an individual human donor, bars are plotted as mean values ± SEM; *n* = 6 donors. Data were analyzed for statistical differences using a one-way ANOVA adjusted for multiple comparisons (Sidak). Asterisks indicate *P* values representing statistical differences as follows: *, *P* < 0.05; **, *P* < 0.01; ***, *P* < 0.001; ****, *P* < 0.0001.

We next wanted to determine if infection of DCs by S. aureus impaired the activation of CD4^+^ T cells. DCs were infected with opsonized wt or Δ*lukAB* USA300 cells for 3 h. Afterward, the extracellular bacteria were killed with gentamicin overnight. The following day, CFSE-labeled autologous CD4^+^ T cells were added to the DCs and cocultured for 3 additional days. The activation and proliferation of lymphocytes was determined as described for Fig. 5C. First, we characterized the state of DCs at 30 min, 3 h, and overnight postinfection in regard to membrane damage and surface levels of proteins involved in migration/phagocytosis (CD11c), antigen presentation (CD1c and HLA-DR), and costimulation of T cells (CD83 and CD86) (32–37). In addition to the clear results seen with the live and dead cell populations after infection with S. aureus (i.e., negligible and maximum staining, respectively), we observed a third population that exhibited an intermediate level of membrane damage staining (Fig. 6A). During wt infection, the population level of the cells with negligible membrane damage began to decrease significantly in comparison to the Δ*lukAB*-infected population and continued to be significantly lower than the Δ*lukAB*-infected population level after overnight treatment with gentamicin (Fig. 6B). At the same time points, we observed an increase in the population of cells exhibiting intermediate staining for membrane damage during wt infection compared to Δ*lukAB* infection (Fig. 6B). In addition to increased cell death, flow cytometry analyses of cells in the gates corresponding to low and intermediate staining revealed a decrease in the levels of CD11c and the antigen-presenting molecules CD1c and HLA-DR as well as in the levels of the costimulatory CD83 and CD86 molecules on the surface of DCs after infection with wt bacteria compared to Δ*lukAB* bacteria (Fig. 6C).

Consistent with the effects of LukAB on DC viability and surface levels of antigen-presenting and costimulatory proteins, CD4^+^ T cell and DC coculture experiments revealed that the DCs infected with wt USA300 exhibited a LukAB-dependent dampening of CD4^+^ T cell stimulation as assayed by cell division (Fig. 6D) and CD25 upregulation (Fig. 6E).

## DISCUSSION

S. aureus commonly causes recurrent infections in the human host without the development of sterilizing immunity (8), suggesting that this bacterium is able to thwart the host immune response. Among the different immune cells involved in the host response to infection, DCs form the bridge between the innate and adaptive immune responses and are crucial for the effective development of immunological memory. Therefore, a comprehensive understanding of the human DC-S. aureus interaction and of the mechanisms that S. aureus employs to subvert DCs is required. Here we demonstrated that S. aureus (both MSSA and MRSA) targets human DCs through the activity of LukAB toxin, resulting in an impaired T cell response.

Most adults harbor S. aureus-specific antibodies (38, 39). Evaluation of children with invasive S. aureus infection revealed that production of LukAB-specific antibodies was enhanced by infection, underlining both the activation of adaptive immunity and the production of this leukocidin during infection (40, 41). Nonetheless, prior exposure to S. aureus does not result in sterilizing immunity or complete protection as evidenced by the frequency of recurrent infections in humans (8). Human S. aureus-specific T lymphocyte responses have been detected (42–45), and T lymphocyte deficiencies are associated with increased susceptibility to S. aureus infections (46). Taking the data together, those studies suggested that adaptive immunity provides some but not sufficient protection against S. aureus reinfection. Our results suggest that by killing DCs and/or dampening the activity of critical surface molecules involved in antigen presentation and costimulation via LukAB, S. aureus is likely to impair the development of immunological memory or recall responses against S. aureus, which ultimately could result in incomplete protection against infection.

While DCs exhibit poor direct antimicrobial activity against S. aureus compared to neutrophils and macrophages (47), these cells can influence immune responses by promoting effector functions of other phagocytes (48). Moreover, these sentinel cells can contribute to protection against S. aureus by activating innate immune mechanisms (49, 50), resulting in “innate memory responses” (51). Thus, by targeting DCs, LukAB can also hinder additional aspects of the human immune response against S. aureus.

While the data presented here clearly demonstrate the importance of LukAB in inhibiting the functionality of and killing human DCs, the nature of the contribution of this toxin to the subversion of the development of adaptive immunity during *in vivo* infection remains to be defined. Although murine models have provided extremely valuable information about anti-S. aureus immune responses, including the involvement of DCs (48, 52, 53), the species specificity exhibited by LukAB and many of the other virulence factors produced by S. aureus limits the utility of these models to unravel the role of these virulence factors during infection (54). Before *in vivo* experiments can be performed, fully susceptible models of infection need to be generated so that the activities of all the relevant leukocidins can be evaluated. This could be achieved by humanizing the relevant toxin receptors (10) or by studying humanized mice (55, 56).

Taking the data together, this report has advanced our understanding of how S. aureus subverts the human immune response to promote infection. Specifically, we identified LukAB as a potent toxin that targets and injures human DCs, highlighting a previously unknown immunosuppressive strategy employed by this pathogen. Ultimately, this knowledge could provide insight into strategies for the development of effective treatments and vaccines against S. aureus infection.

## MATERIALS AND METHODS

### Cell isolation and generation of DCs.

Primary human polymorphonuclear leukocytes (PMNs) and peripheral blood mononuclear cells (PBMCs) from anonymous, healthy donors (New York Blood Center) were isolated from buffy coats as previously described (18). PBMCs were suspended in RPMI 1640 medium (Corning) supplemented with 10% heat-inactivated fetal bovine serum (FBS; Gemini Bio-Products) and 10 mM HEPES (Corning). To generate monocyte-derived dendritic cells, monocytes were purified from total PBMCs by plastic adherence in a 40-ml volume at a concentration of 7 × 10^6^ cells/ml in 150-cm^2^ tissue culture flasks (Corning). After collection of the nonadherent fraction and washing of the adherent cells with RPMI medium, cells were cultured for 4 days at 37°C and 5% CO_2_ in RPMI medium supplemented with 10% FBS, 10 mM HEPES, 100 U/ml penicillin, 100 μg/ml streptomycin, 110 U/ml granulocyte-macrophage colony-stimulating factor (GM-CSF) (Leukine; Sanofi), and 282 U/ml interleukin-4 (IL-4) (Affymetrix, eBioscience). The medium was replenished with fresh IL-4 and GM-CSF every 2 days. The nonadherent monocyte-depleted PBMCs were frozen in 10% dimethyl sulfoxide (DMSO)–40% FBS–RPMI medium until use in T cell proliferation assays.

### Bacterial strains and culture conditions.

The S. aureus isolates used in this study are listed in Table 1. S. aureus USA300 strain AH-LAC (19) was used in all experiments as the wild-type (wt) strain unless otherwise indicated. Bacteria were routinely grown at 37°C on tryptic soy agar (TSA). Overnight cultures were grown in 5 ml tryptic soy broth (TSB) in 15-ml tubes under shaking conditions at 180 rpm with a 45° angle. A 1:100 dilution of overnight culture was subcultured into RPMI medium (Invitrogen) supplemented with 1% Casamino Acids (RPMI medium–Cas; pH 7.3) and incubated for another 4.5 h before being used for infection. When appropriate, chloramphenicol was supplemented to the media at a final concentration of 10 µg/ml. The pOS1-*P_sarA_*-*SOD*-*RBS*-sGFP plasmid (57) was transformed to the panel of AH-LAC isogenic mutant strains.

### Exoprotein isolation and immunoblotting.

S. aureus was cultured as described above and normalized to the same optical density at 600 nm (OD_600_). Bacterial cells were pelleted by centrifugation at 4,000 rpm for 10 min. Proteins in the supernatants were filtered through a 0.2-µm-pore-size filter and precipitated with 10% (vol/vol) trichloroacetic acid (TCA) at 4°C. The precipitated proteins were washed with 100% ethanol, air-dried, resuspended with 8 M urea and 2× SDS loading buffer, and boiled. Proteins were separated on 12% SDS-PAGE gels, transferred to nitrocellulose membranes, and probed sequentially with rabbit anti-LukA (20) (1:5,000), rabbit anti-LukB (20) (1:1,000), and rabbit anti-Hla (Sigma) (1:5,000) polyclonal antibodies. Alexa Fluor 680-conjugated goat anti-rabbit IgG (Life Technologies) (1:25,000) was used as a secondary antibody in a mixture with phosphate-buffered saline (PBS; Corning) supplemented with 0.1% Tween 20. Membranes were scanned using an Odyssey Clx imaging system (Li-Cor Biosciences).

### Infection assays.

DCs were seeded in 96-well round-bottom plates at 1 × 10^5^ cells per well in a final volume of 100 µl of RPMI medium without phenol red (Gibco) that was supplemented with 0.05% human serum albumin (Seracare) and 10 mM HEPES. DCs were infected with S. aureus at a multiplicity of infection (MOI) of 25, 10, 5, 1, or 0.1 and incubated at 37°C under shaking conditions at 180 rpm for 15 min, 1 h, 2 h, or 4 h. To assess the cell death pathways, DCs were incubated with one of the inhibitors necrostatin-1 (Nec-1; Enzo Life Sciences), necrosulfonamide (NSA; Calbiochem), GSK-872 (Calbiochem), VX-765 (Calbiochem), Ac-DEVD-CHO (Enzo Life Sciences), or Z-VAD-FMK (Selleck Chemicals) for 30 min at 37˚C and 5% CO_2_ without shaking prior to infection with S. aureus. Following infection, cells were pelleted by centrifugation at 1,500 rpm at 4°C for 5 min and lactate dehydrogenase (LDH) release was measured using the CytoTox-ONE homogeneous membrane integrity assay (Promega). In brief, 25 µl of culture supernatant was mixed with 25 µl of LDH reagent and incubated for 15 min at room temperature (RT). Fluorescence was measured using a PerkinElmer 2103 Envision multilabel plate reader (excitation, 555 nm; emission, 590 nm) and normalized to wells containing cells without S. aureus (0% cell lysis) and cells with 0.05% Triton X-100 (100% cell lysis).

### Cytotoxicity assays.

To analyze the susceptibility of human PMNs and DCs to S. aureus leukocidins, cells were seeded at 1 × 10^5^ cells per well in RPMI medium without phenol red (Gibco) supplemented with 10% FBS and incubated with recombinant S. aureus leukocidins for 1 h at 37°C and 5% CO_2_. After incubation, the supernatants were analyzed for the presence of LDH using the CytoTox-ONE homogeneous membrane integrity assay as described above. To determine the membrane damage of DCs and CD4^+^ T cells after 24 h exposure to leukocidins, cells were stained with eFluor 450 fixable viability dye (eBiosciences) and analyzed by flow cytometry (Cytoflex; Beckman Coulter).

### Luminescence reporter assays.

To measure leukocidin promoter activity in S. aureus during DC interactions, bacteria containing reporter plasmids (individual leukocidin promoters fused to the luciferase operon from Photorhabdus luminescens present in the pXEN plasmid [Xenogen]) (11) were used. For each experiment, bacteria were freshly streaked onto TSA plates supplemented with 10 μg/ml chloramphenicol and bacteria were grown overnight in 5 ml tryptic soy broth (TSB; BD Difco) supplemented with 10 μg/ml chloramphenicol at 37°C under shaking conditions (180 rpm). The following day, bacteria were first subcultured 1:100 in fresh RPMI medium–Cas for 3 h and subsequently subcultured 1:10 for another 3 h in fresh RPMI medium–Cas to reduce background luminescence. Cells and bacteria were coincubated at an MOI of 10 at 37°C under shaking conditions at 180 rpm. Luminescence readings were taken at time points from time point zero to 4 h using a PerkinElmer Envision 2103 multilabel reader. Values corresponding to the background fluorescence of medium alone were subtracted from the readings determined for the samples.

### CD11b detection.

PMNs and DCs were stained with anti-CD11b(activated)-APC monoclonal antibody (MAb) CBRM1/5 and anti-CD11b (total)-brilliant violet 605 MAb M1/70 (BioLegend). After washing, samples were fixed with PBS supplemented with 2% FBS, 2% paraformaldehyde, and 0.05% sodium azide and analyzed by flow cytometry (Cytoflex; Beckman Coulter). Data were analyzed using FlowJo software.

### Bacterium internalization assays.

S. aureus containing the pOS1-*P_sarA_*-*SOD*-*RBS*-sGFP plasmid was opsonized by incubation with 10% freshly isolated human serum, prepared as previously described (58), for 30 min at 37°C under shaking conditions. Nonopsonized bacteria were treated similarly but were incubated in media instead of human serum. After incubation, bacteria were washed twice, suspended in RPMI medium–0.05% HSA–10 mM HEPES, and diluted to infect DCs at an MOI of 10. To prevent bacterial uptake, cytochalasin D was used during infection at a final concentration of 10 μg/ml. To measure bacterial uptake, DCs and bacteria were incubated for 15 min at 37°C under shaking conditions, after which samples were fixed using PBS supplemented with 2% FBS, 2% paraformaldehyde, and 0.05% sodium azide. Bacterial uptake was assessed by flow cytometry analysis, determining the green fluorescent protein (GFP) levels of the DCs. In parallel, DC lysis was assessed by measuring the release of LDH (as described above) after incubation of DCs and bacteria for 2 h at 37°C under shaking conditions.

### Microscopy.

To image the uptake of bacteria by DCs, the leukocidin-lacking S. aureus mutant (Δ*hlgACB* Δ*lukED* Δ*pvl* Δ*lukAB*) was transformed with the pOS1-*P_sarA_*-*SOD*-*RBS*-sGFP plasmid (VJT 49.34). These bacteria were subcultured for 4.5 h in TSB–10 μg/ml chloramphenicol and subsequently opsonized in fresh human serum as described above (see “Bacterium internalization assays”). For time-lapse microscopy, 200 μl of 1 × 10^5^/ml DCs were seeded in RPMI medium–0.05% HSA–10 mM HEPES in chambered coverglasses (Nunc; 8-well Lab-Tek chambered coverglass, catalog no. 155411) and kept at 37°C and 5% CO_2_ until microscopy analysis (∼3 h). At the microscope (Nikon Eclipse Ti; 60× numerical aperture [NA], 1.4 phase lens, Andor Zyla camera), a 50-μl volume of the preopsonized bacteria was added at 5 × 10^6^ CFU/ml (MOI of 10) to the cells and imaged using phase contrast to delineate DCs and fluorescence to record GFP-expressing bacteria. To verify that the bacteria were completely internalized (rather than sticking to the exterior of the DCs), fixed and labeled samples were imaged using a VT-iSIM instant structured illumination microscope (VisiTech International; Leica 63× NA, 1.4 lens), which behaves similarly to a spinning disk confocal microscope, to collect diffraction-limited optical sections through the DCs. For this analysis, 25 μl of 5 × 10^7^/ml bacteria was incubated with 25 μl of DCs (5 × 10^6^/ml) and 50 μl of 2% fresh human serum in a round-bottom 96-well plate for 15 min at 37°C under shaking conditions. After incubation, samples were fixed with 2% paraformaldehyde overnight at 4°C. The following day, samples were washed with Hanks’ balanced salt solution (HBSS; Corning) and incubated with 5 μg/ml wheat germ agglutinin-Alexa Fluor 594 (WGA-AF594; Thermo Fisher Scientific) for 15 min at room temperature (RT) in HBSS to stain the DCs. The samples were washed in HBSS and seeded in a chambered coverglass (∼ 2.5 × 10^4^ DCs/well) for microscopy analysis.

### DC-CD4^+^ T lymphocyte coculture assays.

Autologous CD4^+^ T lymphocytes were purified from the monocyte-depleted fraction using an EasySep human CD4^+^ T cell enrichment kit (Stem Cell Technologies) according to manufacturer’s instructions. Cells were typically >97% CD3^+^ CD4^+^ as determined by flow cytometry using anti-CD3-PE (phycoerythrin) Cy7 MAb UCHT1 and anti-CD4-Pacific Blue MAb OKT4 (BioLegend). CD4^+^ T cells were then labeled with CFSE (CFSE cell division tracker kit; BioLegend) according to the manufacturer’s instructions. In short, 10 × 10^6^ to 100 × 10^6^ cells/ml were incubated at a concentration of 5 μM CFSE in PBS for 10 min in the dark at 37°C and 5% CO_2_, after which the staining was quenched by washing the cells twice in ice-cold RPMI medium–10% FBS. CD4^+^ T cells and autologous DCs were plated in a TC-treated 96-well round-bottom plate at a 10:1 ratio with 50,000 CD4^+^ T cells and a total volume of 100 μl per well. Samples were plated in duplicate and were pooled on the day of analysis. For experiments using bacterial supernatant as a stimulus, a 0.01% concentration of supernatant from S. aureus strain Newman, which lacks all the leukocidins and alpha-toxin (ΔΔΔΔΔ Newman; Δ*lukED* Δ*hlgACB::tet* Δ*lukAB::spec* Δ*hla::ermC*) and naturally lacks PVL (59), and purified leukocidins were added to the wells. After 3 days of coculture, samples were stained with anti-CD3-PECy7 MAb UCHT1, anti-CD4-Pacific Blue MAb OKT4, and anti-CD25-APC MAb BC96 (BioLegend). Samples were fixed by using PBS–2% FBS–2% paraformaldehyde–0.05% sodium azide. The CD4^+^ T lymphocytes were analyzed by flow cytometry for CFSE dilution and the presence of CD25.

In the assays where the DCs were infected with S. aureus, the DCs were preinfected statically with opsonized S. aureus at an MOI of 5 at 37˚C and 5% CO_2_. After 3 h of infection, the cells were treated with 50 μg/ml gentamicin and 1× penicillin-streptomycin (Corning catalog no. 30-002-CI) to kill extracellular bacteria and incubated overnight at 37°C and 5% CO_2_. The next day, autologous CD4^+^ T lymphocytes were isolated, labeled with CFSE, added to the preinfected DCs, and cocultured for 3 days. Flow cytometry analysis was performed using a Cytoflex (Beckman Coulter), and data were analyzed using FlowJo software.

### Measurement of infection effects on DCs.

To determine the state of DCs before coculturing with T cells was performed, DCs were infected with S. aureus for 30 min or 3 h or overnight as described for the DC-CD4^+^ T lymphocyte coculture assays. At each time point, DCs were washed, treated with Fc receptor blocking solution (human TruStain FcX; BioLegend), and then stained with anti-human CD11c-PerCP (peridinin chlorophyll protein) MAb Bu15, anti-human CD1c-Pacific Blue MAb L161, anti-human HLA-DR-APC/Cy7 MAb L243, anti-human CD83-PE MAb HB15e, and anti-human CD86-APC MAb IT2.2 or corresponding isotype controls (BioLegend). After washing, DCs were stained with Zombie Red fixable viability dye (BioLegend) to determine the level of membrane damage. The samples were fixed with PBS supplemented with 2% FBS, 2% paraformaldehyde, and 0.05% sodium azide and analyzed by flow cytometry (Cytoflex; Beckman Coulter). Data were analyzed using FlowJo software.
